# Low-Dose Initiations of Buprenorphine in the Fentanyl Era—A Search for Evidence-Based Approaches to an Evolving Crisis

**DOI:** 10.1001/jamanetworkopen.2024.56261

**Published:** 2025-01-02

**Authors:** Justin Berk, Michael Rose, Ashish P. Thakrar

**Affiliations:** Department of Medicine, Alpert Medical School at Brown University, Providence, Rhode Island; Department of Pediatrics, Alpert Medical School at Brown University, Providence, Rhode Island; Department of Medicine, Johns Hopkins University School of Medicine, Baltimore, Maryland; Department of Medicine, University of Pennsylvania Perelman School of Medicine, Philadelphia

As fentanyl has become dominant in the US drug supply, clinicians and patients have sought alternative strategies for buprenorphine initiation to avoid precipitated withdrawal. Limited evidence on the effectiveness of alternative strategies leaves a noticeable absence of guidelines on best practices on how to start buprenorphine treatment for individuals with opioid use disorder in the fentanyl era, particularly in the outpatient setting. Suen et al^4^ sought to address this gap. Using a retrospective cohort analysis, the authors compared 2 low-dose initiation (LDI) protocols to start buprenorphine in patients using fentanyl. The results: we still have a long way to go.

Prior to the fentanyl era, initiation of buprenorphine through traditional protocols was relatively simple and effective. Barriers to treatment were less clinical and more policy based (eg, X waiver) or logistic (eg, pharmacy availability). Today, the X waiver has been abolished and access to buprenorphine is increasing nationwide, but fentanyl has elevated the concern surrounding buprenorphine-precipitated withdrawal (ie, the rapid onset of withdrawal symptoms associated with taking buprenorphine too soon after using a full-agonist opioid, such as fentanyl).

The true prevalence of precipitated withdrawal remains unclear. Recent literature has identified that approximately 1 in 8 buprenorphine initiations in hospital settings is associated with precipitated withdrawal from fentanyl, although other studies have found far higher^1^ and lower^2^ estimates. Regardless of the prevalence, the concern for precipitated withdrawal is substantial. Patients not only endure severe symptoms but may also be deterred from future lifesaving treatment with medications for opioid use disorder.

LDI with opioid continuation involves starting with low doses of buprenorphine and increasing over several days while continuing other opioids, in an effort to avoid precipitated withdrawal. There are now more than 2 dozen case series or retrospective cohort studies describing LDI, although most data come from inpatient settings without control groups.^3^

Suen et al^4^ compared 2 outpatient LDI protocols: a 4-day vs 7-day approach. The authors used the electronic health record to retrospectively review 175 LDI attempts among 126 patients in 2 clinics in San Francisco, California. The cohort was young (median [IQR] age, 35 [29-44] years) and predominantly male (90 [71%]), with 85 individuals (67%) using methamphetamine, 99 (79%) predominantly smoking fentanyl, and 87 (71%) with prior experience with buprenorphine. Choice of LDI approach (vs high-dose initiation with ≥8 mg buprenorphine after 48-72 hours of abstinence) was made collaboratively during routine clinical care. All patients who chose LDI did so because they wanted to avoid precipitated withdrawal.

Overall, Suen et al^4^ found that only 34% of patients who started LDI completed buprenorphine initiation and picked up a buprenorphine maintenance prescription, with no difference detected between the 4-day vs 7-day protocol. At 28 days, only 22% of patients remained in buprenorphine treatment, again with no difference between treatment groups.

Given that only 1 in 3 patients ultimately finished buprenorphine initiation, it might be tempting to impugn all LDI protocols based on these findings. But it is important to contextualize these results. First, both the sample size and study population in this study may limit generalizability to other settings or regions. The cohort had high rates of fentanyl inhalation, co-occurring methamphetamine use, and unstable housing. Thus, these findings might not represent outcomes among people with different drug use patterns or those with more stable social situations. Second, these results may not apply to inpatient LDI, where retrospective cohort studies have found much higher success rates, possibly because full-agonist opioids can be directly administered on a more regular schedule.^5^ Third, it is unknown how these outcomes may compare with high-dose or traditional initiation outcomes in similar patients in the study.

Despite these limitations, LDI, when successful, can still enable individuals to start lifesaving treatment. This study fills a gap in our understanding of LDI since it is one of the first to assess outcomes among people using fentanyl in an outpatient setting where patients were instructed to continue drug use. By highlighting the challenge of starting buprenorphine in the current era, Suen et al^4^ provide important insights and guidance for future research and clinical practice.

## Next Steps: A Research Agenda

A research agenda can build on these results to continue moving the field forward. First, prioritize clinical trials. Suen et al^4^ highlighted an urgent need for prospective, randomized, and/or blinded clinical trials that evaluate buprenorphine initiation and retention strategies for people who use fentanyl. The lack of evidence for basic treatment nearly 7 years into the fentanyl crisis is glaring. Funding agencies should continue to prioritize such research.

Second, use new approaches to research design. Novel methodologies can build on traditional clinical trials and work to provide clinical insights. Trial emulation studies, adaptive trial designs, and partially randomized preference trials seek to address limitations in feasibility and ethical concerns by simulating randomization or incorporating patient preferences, thus enhancing the applicability of findings to clinical settings and supporting more personalized approaches to addiction treatment.

Third, characterize risk factors. We must identify and examine risk factors for buprenorphine-precipitated withdrawal and treatment retention. Treatment protocols may not generalize to all regions, drug use patterns, and individuals. By better understanding risks, clinicians can provide patient-specific approaches.

Fourth, explore cotreatment of stimulant use disorder. Given the high prevalence of co-occurring methamphetamine use in this study sample (with similar patterns nationally), future studies should investigate cotreatment of stimulant use disorders. Some preliminary data highlight how stimulant treatment may improve buprenorphine treatment retention.^6^

A challenge remains for patients and clinicians who must make decisions about how to start buprenorphine today, while awaiting further evidence. Outpatient LDI may serve as one way to support patients who fear precipitated withdrawal and want to continue drug use during the initiation process. Some of the fear may be overstated: hospital^2^ and emergency department^7^ studies, as well as survey studies based on outpatient self-report,^1^ indicate that traditional buprenorphine initiation does not trigger precipitated withdrawal for most patients, even in those using fentanyl. However, these studies did not evaluate treatment retention. Data from Suen et al^4^ suggest that avoiding precipitated withdrawal with LDI does not seem to translate into a robust treatment retention rate. Thus, perhaps LDI is not yet ready to replace traditional initiation as the standard of care in outpatient settings.

In individuals who have not tolerated or who decline traditional buprenorphine initiations, it may be necessary to consider alternative initiation approaches, such as LDI or high-dose buprenorphine initiation or methadone. However, as the findings from Suen et al^4^ suggest, until further evidence accumulates, we should be cautious about recommending widespread adoption of any specific alternative as the default initiation strategy.

It is also crucial to counsel patients about the unknown rates of success of these novel approaches. While efforts like those by Suen et al^4^ work to fill the current knowledge gaps, we must work to ensure these gaps do not lead to an even more discouraging result: the erosion of trust from a stigmatized population that already has considerable distrust for the medical system. Building trust is not just a goal but a necessity to advance equitable care and to ensure new evidence (such as this study) is applied to improve outcomes for those struggling with addiction.
